# Contribution of the Endosomal-Lysosomal and Proteasomal Systems in Amyloid-β Precursor Protein Derived Fragments Processing

**DOI:** 10.3389/fncel.2018.00435

**Published:** 2018-11-22

**Authors:** Caroline Evrard, Pascal Kienlen-Campard, Mathilde Coevoet, Rémi Opsomer, Bernadette Tasiaux, Patricia Melnyk, Jean-Noël Octave, Luc Buée, Nicolas Sergeant, Valérie Vingtdeux

**Affiliations:** ^1^Université de Lille, Inserm, Centre Hospitalier-Universitaire de Lille, UMR-S 1172 – Centre de Recherche Jean-Pierre Aubert, Lille, France; ^2^Alzheimer Research Group, Institute of Neuroscience, Université catholique de Louvain, Brussels, Belgium

**Keywords:** Alzheimer’s disease, amyloid precursor protein, amyloid beta peptide, APP-CTF, C99

## Abstract

Aβ peptides, the major components of Alzheimer’s disease (AD) amyloid deposits, are released following sequential cleavages by secretases of its precursor named the amyloid precursor protein (APP). In addition to secretases, degradation pathways, in particular the endosomal/lysosomal and proteasomal systems have been reported to contribute to APP processing. However, the respective role of each of these pathways toward APP metabolism remains to be established. To address this, we used HEK 293 cells and primary neurons expressing full-length wild type APP or the β-secretase-derived C99 fragment (β-CTF) in which degradation pathways were selectively blocked using pharmacological drugs. APP metabolites, including carboxy-terminal fragments (CTFs), soluble APP (sAPP) and Aβ peptides were studied. In this report, we show that APP-CTFs produced from endogenous or overexpressed full-length APP are mainly processed by γ-secretase and the endosomal/lysosomal pathway, while in sharp contrast, overexpressed C99 is mainly degraded by the proteasome and to a lesser extent by γ-secretase.

## Introduction

**FIGURE 1:**
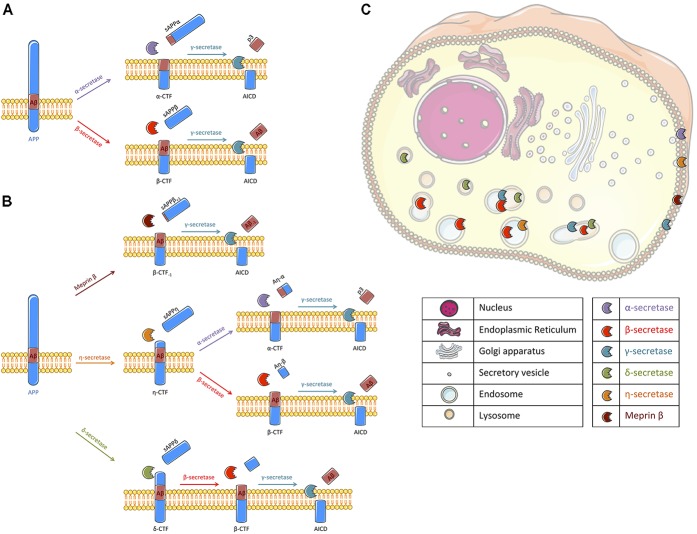
Schematic representation of APP processing and the cellular localization of the different secretases involved. **(A)** Following synthesis and maturation, the amyloid precursor protein (APP) can classically follow two different pathways: the non-amyloidogenic or the amyloidogenic pathways, the latest being at the origin of Aβ peptides production. In the non-amyloidogenic pathway, APP is first cleaved by α-secretase (*mallow*) within the Aβ sequence, liberating a soluble fragment sAPPα and a transmembrane carboxy-terminal fragment (APP-CTF) called α-CTF. Then, γ-secretase (*turquoise*) processes α-CTF into p3 and APP intracellular domain (AICD). In the amyloidogenic pathway, sAPPβ and β-CTF are released following APP cleavage by β-secretase (*red*). β-CTF is further processed by γ-secretase into Aβ peptide and AICD. **(B)** Alternatively to a direct cleavage by α- or β-secretase, APP can be processed by other secretases that have been recently described. Meprin β (*brown*) can act as an alternative β-secretase releasing sAPPβ with 1 amino acid longer and β-CTF with an amino acid less. β-CTF_-1_ is similarly cleaved by γ-secretase. η-secretase (*orange*) cleaves APP into sAPPη and η-CTF. η-CTF can follow either the amyloidogenic or the non-amyloidogenic pathway, releasing Aη-β and β-CTF or Aη-α and α-CTF, respectively. The remaining CTFs (α- and β-) are likely processed by γ-secretase. δ-secretase (*green*) processes APP into sAPPδ and δ-CTF. Cleavage of δ-CTF by β- and γ-secretase releases Aβ and AICD. **(C)** All these secretases are active in different cellular compartments. Thus, we propose a schematic representation of their spatial location for APP cleavage in agreement with the literature. α-secretase and meprin β were described to cleave APP at the cell surface where α-CTF and β-CTF_-1_ can then be processed by γ-secretase (Lichtenthaler, 2011; Jäckle et al., 2015; Wang et al., 2017). γ-secretase cleavage was reported to occur at the plasma membrane and in endosomes (Kaether et al., 2006). β-secretase processing of APP is reported to occur in endosomal/lysosomal acidic compartments as it is the case for the δ-secretase (Vassar et al., 1999; Zhang et al., 2015) where APP-CTFs could also be cleaved by γ-secretase. The location of APP processing by η-secretase is still unclear but it is likely that η-secretase is located at the plasma membrane and also improves APP localization to endosomes (Baranger et al., 2016a, 2017). Figure was produced in part using Servier Medical Art.

Amyloid deposits, that mainly consist of the extracellular aggregation of amyloid β-peptides (Aβ) into plaques, are one of the major pathological hallmarks of Alzheimer’s disease (AD) and the main targets of current clinical trials (Hung and Fu, 2017). Aβ peptides are generated by sequential and compartmentalized cleavages of the type I transmembrane protein named Amyloid Precursor Protein (APP). Proteolytic cleavage of APP by β-secretase (mainly BACE1), characterized as the first step of the amyloidogenic pathway, releases APP carboxy-terminal fragments (APP-CTFs) named β-CTF or C99 that remain membrane bound and the soluble sAPPβ fragments that are secreted. β-CTFs are then processed within their transmembrane domain by γ-secretase, a transmembrane proteolytic complex, thereby releasing Aβ peptides and APP Intracellular Domain (AICD) (for a review see Wang et al., 2017). Alternatively to this amyloidogenic pathway, APP can be processed by an α-secretase activity (Vingtdeux and Marambaud, 2012), which cleaves within the Aβ sequence thus precluding its production. This α-secretase cleavage of APP releases soluble sAPPα fragments that are secreted and membrane-bound APP-CTFs named α-CTFs or C83, that can be further processed by γ-secretase (Figure 1A). Recently, the identification of new secretases, like δ-secretase (asparagine endopeptidase), η-secretase (MT5-MMP) and meprin β, have added to the complexity of APP processing (Andrew et al., 2016). Meprin β can be considered as an alternate β-secretase enzyme since it gives rise to a β-CTF starting at position 2 (Bien et al., 2012; Becker-Pauly and Pietrzik, 2017). δ -secretase and η-secretase generate larger APP-CTFs that can be further processed by α- or β- and γ-secretases (Willem et al., 2015; Zhang et al., 2015; Baranger et al., 2016b; Figure 1B). Finally, it is also important to note that these secretases are active at various intracellular locations. While their exact location is still debated, we propose a schematic representation of their spatial location for APP cleavage in agreement with the literature (Figure 1C).

Among all the fragments generated along APP processing, β-CTFs are of particular interest since they are the direct precursors of Aβ peptides. In addition, β-CTFs have been shown to accumulate before Aβ peptides *in vivo*, and suggested to be instrumental in the initiation of the neurodegenerative process and cognitive alterations (Lauritzen et al., 2012). Inhibition of β-secretase cleavage of APP rescues long-term potentiation (LTP) and memory deficits in a mouse model of AD (Tamayev et al., 2012). Of note, β-CTFs accumulation was also described to occur in AD brains (Pera et al., 2013). While the exact impact of β-CTFs accumulation in AD is not completely elucidated yet, a better understanding of the mechanisms involved in APP-CTFs, and in particular β-CTFs, degradation is needed.

Indeed, alternatively to their cleavage by γ-secretase, lysosomal proteases and/or the proteasome are involved in APP-CTFs degradation. APP has an internalization carboxy-terminal NPxY motif and is thus found along endosomal/lysosomal compartments (Morel et al., 2013; Tam et al., 2016; Xu et al., 2017). Inhibition of lysosomal proteases induces an accumulation of APP-CTFs (Vingtdeux et al., 2007a,b; González et al., 2017), showing that the endosome/lysosome pathway is important for the processing of APP. Other reports suggested that APP-CTFs, in particular β-CTFs, were rather processed by the proteasome (Skovronsky et al., 2000; Nunan et al., 2001, 2003). Additionally, the proteasome was also suggested to indirectly modulate APP metabolism by regulating the degradation of proteases implicated in APP metabolism (Marambaud et al., 1997; Flood et al., 2005).

Although γ-secretase, proteasome and lysosomal proteases were shown to be involved in APP processing and β-CTFs degradation, their mutual implication remains to be established. In the present study, we compared the contribution of these three degradation pathways on APP processing in HEK cells and in primary neuronal cultures expressing either full-length APP^WT^ or the C99 fragment by using selective pharmacological drugs (Dyrks et al., 1992b).

## Materials and Methods

### HEK Cell Culture and Primary Neuronal Cell Culture

HEK293 stably expressing or not full-length APP^WT^ or C99 were previously described (Lefranc-Jullien et al., 2005) and kindly provided by Prof. F. Checler. Briefly, HEK cells were grown in Dulbecco’s Modified Eagle Medium (DMEM, high glucose, pyruvate – GIBCO by Life Technologies) supplemented with 10% fetal bovine serum, 2 mM L-glutamine, 1 mM non-essential amino-acids and penicillin/streptomycin (GIBCO by Life Technologies) in a 5% CO_2_ humidified incubator at 37°C. Primary cultures of rat embryonic cortical neurons were prepared as previously described. After 6 days of culture, neurons were infected with recombinant adenovirus expressing either APP^WT^ (corresponding to wild-type APP695 isoform) or C99 as described (Kienlen-Campard et al., 2002; Pitsi et al., 2002). All animal procedures used in the study were carried out in accordance with institutional and European guidelines and experimental protocols were approved by the Animal Ethics Committee from the Université catholique de Louvain (UCL, Brussels, Belgium).

### Drug Treatments

Bafilomycin A1 (Baf_A1_) and Compound E (CompE) were purchased from Merck Millipore. Chloroquine (CQ), Lactacystin (Lacta) and MG132 were purchased from Sigma-Aldrich. Epoxomicin (Epoxo) was purchased from Abcam. Cells were plated into 6-well plates at a density of 500,000 cells per well, 24 h (h) before drug exposure. Cultures were briefly washed once with warm phosphate-buffered saline (PBS) and then exposed for 6 or 24 h to drug-treatments at the indicated concentrations. For primary cultures, treatments were performed at day 4 post-infection. At the end of treatments, the conditioned medium was collected and kept at -80°C until use to evaluate the levels of Aβ_1-40_, Aβ_1-42_ peptides and sAPPα/sAPPβ secretion by ELISA and electro-chemiluminescence immunoassay, respectively. Then, cells were rinsed once with PBS and collected in 100 μL of Laemmli buffer (10 mM Tris, 20% glycerol and 2% Sodium dodecyl sulfate) using a cell-scraper. The lysate was sonicated for 5 min before measurement of total protein concentration using the Pierce BCA Protein Assay Kit (Thermo scientific) according to the manufacturer’s protocol. Samples were conserved at -80°C before analysis.

### Subcellular Fractionation

HEK293 stably expressing or not full-length APP^WT^ or C99 were retrieved into 1 mL of a buffer composed of 10 mM Tris–HCl, pH = 7.5; 0.25 M sucrose; 1 mM EDTA, 1× complete protease inhibitor cocktail and then, passed through a 26 gauge needle 10 times. Obtained lysates were centrifuged at 500 g for 10 min and pellets were discarded. Protein concentration was determined using the Pierce BCA Protein Assay Kit (Thermo scientific) according to the manufacturer’s protocol. Supernatants were adjusted to 1.5 μg/μL and an aliquot (before ultra-centrifugation) was kept as the total protein fraction. Then, supernatants were centrifuged at 120,000 *g* for 45 min at 4°C to separate cytosolic (supernatants) and membrane fractions (pellets). Membrane fractions were resuspended in 250 μL of NuPage NuPAGE^®^ lithium dodecyl sulfate (LDS) 2X sample buffer supplemented with 20% NuPAGE^®^ sample reducing agents (Invitrogen). Total fraction, cytosolic and membrane fractions were analyzed by western blot.

### Western Blotting

Cell protein samples were prepared for western-blot analysis by adding 1 volume of NuPage NuPAGE^®^ LDS 2X sample buffer supplemented with 20% NuPAGE^®^ sample reducing agents (Invitrogen). Samples were heated 10 min at 100°C. 10 μg of total proteins per well were loaded onto precast 4–12% Criterion XT Bis-Tris polyacrylamide gels (Bio-Rad) and electrophoresis was achieved by applying a tension of 150 V during 90 min using a Criterion electrophoresis Cell with the NuPAGE^®^ MOPS SDS running buffer (1X). Proteins were transferred to a nitrocellulose membrane of 0.4 μM pore size (G&E Healthcare) using the Criterion blotting system by applying a tension of 100 V for 45 min. To resolve proteins of low molecular weights such as CTFs of APP, equal quantities of total proteins (20 μg/lane) were loaded on 16% Tris-tricine polyacrylamide gels. Tris-tricine SDS-polyacrylamide gel electrophoresis and Western-blotting were performed as previously described (Vingtdeux et al., 2005). Molecular weights calibration was achieved using molecular weight markers (Novex and Magic Marks, Life Technologies). Protein transfer and quality were determined by a reversible Ponceau Red coloration (0.2% Xylidine Ponceau Red and 3% Trichloroacetic acid). Membranes were then blocked in 25 mM Tris–HCl pH 8.0, 150 mM NaCl, 0.1% Tween-20 (v/v) (TBS-T) and 5% (w/v) of skimmed milk (TBS-M) or 5% (w/v) of bovine serum albumin (TBS-BSA) depending on the antibody during 1 h. Membranes were rinsed three-times 10 min in TBS-T before incubation with primary antibodies overnight at 4°C. Membranes were rinsed 3 times 10 min with TBS-T and then incubated with secondary antibodies for 45 min at room temperature (RT). The immunoreactive complexes were revealed using the ECL^TM^ Western Blotting Detection Reagents (G&E Healthcare) and a standard ECL detection procedure was then performed. Quantifications of protein expression levels were performed with ImageJ Software (NIH).

### Antibodies

APP and APP-CTFs were detected with a rabbit APP-Cter-C17 antibody (1/5,000) that was raised against the last 17 amino acids of the human APP sequence (Sergeant et al., 2002; Vingtdeux et al., 2005). β-CTFs were detected using an anti-amyloid β antibody clone W02 (1/2,000) obtained from Merck Millipore. Anti-LC3B (1/2,000) and anti-p62/SQSTM1 (1/1,000) were purchased from Cell Signaling Technology. Anti-mono and polyubiquitinylated conjugates (1/1,000) was purchased from Enzo Life Sciences. Anti-N-cadherin (1/1,000) was purschased from BD Biosciences. Anti-β-actin antibody (1/10,000), anti-GAPDH (1/50,000), anti-β-tubulin (1/1,000) and all secondary antibodies coupled with horseradish peroxidase were purchased from Sigma-Aldrich.

### Aβ Peptides Quantification

Collected medium was spun at 200 g for 5 min to eliminate cell debris. Secreted Aβ_1-40_ and Aβ_1-42_ peptides concentrations in pg/mL were determined using amyloid beta 40 and 42 Human ELISA kits (Invitrogen) according to the manufacturer’s instructions.

### sAPPα/sAPPβ Quantification

Conditioned medium collected at the end of experiments was briefly centrifuged as describe above to eliminated cells debris. sAPPα and sAPPβ concentrations were determined using the sAPPα/sAPPβ multiplex kit (Meso Scale Diagnostics, MSD^®^) according to the manufacturer’s instructions.

### Statistical Analysis

The number of experiments for each experimental condition is indicated in the figure legends. Statistical analyses were performed with GraphPad Prism 6 program (GraphPad Software) by using the unpaired Student’s test for pairwise comparisons and One way ANOVA with Bonferroni’s multiple comparisons test for multiple pairwise comparisons. Statistical significance was set at ^∗^*p* < 0.05, ^∗∗^*p* < 0.01, ^∗∗∗^*p* < 0.001, and ^∗∗∗∗^*p* < 0.0001. All data are reported as mean ± standard error of the mean (SEM) of at least *n* = 3 experiments.

## Results

### APP-Derived Carboxy-Terminal Fragments Are Mainly Processed by γ-Secretase and Lysosomal Proteases

**FIGURE 2:**
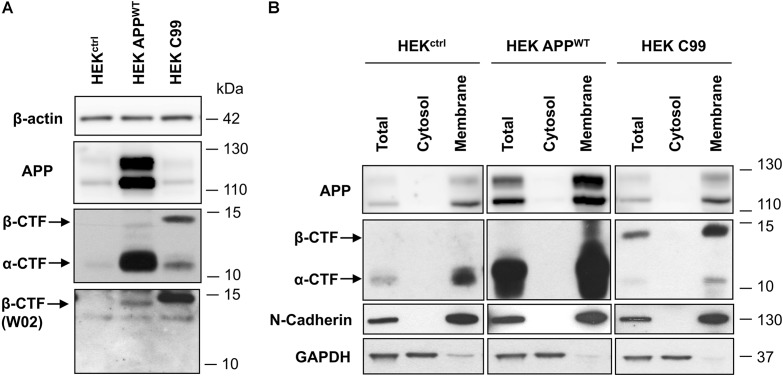
Comparison of APP and C99 expression in the different HEK 293 cell lines used in this study. **(A)** Western-blot analysis of β-actin, total APP, APP-CTFs and β-CTFs (W02) expression in naive HEK 293 cells (HEK^ctrl^) or HEK 293 overexpressing full length APP^WT^ (HEK APP^WT^) or C99 (HEK^C99^). β-actin staining was used as loading control. **(B)** Western-blot analysis of total APP, APP-CTFs, N-cadherin and GAPDH in total lysates, cytosolic and membrane fractions obtained from HEK^ctrl^, HEK APP^WT^, and HEK^C99^. GAPDH staining was used as a marker and loading control for total and cytosolic fractions while N-cadherin was used as a marker and loading control for membrane fractions. See Supplementary Figure S3 for corresponding uncropped images of western-blots.

First, APP and APP-CTFs expression was compared between the cell lines used in this study: control naive HEK (HEK^ctrl^), HEK stably expressing full length wild-type APP (HEK APP^WT^) and HEK cells stably expressing the last 99 carboxy-terminal residues of APP (HEK^C99^) fused to the APP signal sequence and two additional residues from APP 695 (Leu, Glu) (Dyrks et al., 1992a, 1993). Overexpression of APP in HEK APP^WT^ and C99 in HEK^C99^ was confirmed by western-blot analysis (Figure 2A). While in HEK^ctrl^ cells, endogenous total APP and APP-CTFs were barely detected in control conditions, they were both increased in HEK APP^WT^. In HEK^C99^, total APP levels were not affected by C99 overexpression since APP expression is identical to HEK^ctrl^ cells. However, HEK^C99^ cells displayed a strong expression of the β-CTF/C99 fragments. The bands corresponding to β-CTFs derived from APP processing and overexpressed C99 were identified using the anti-amyloid β antibody clone W02 directed againt the Aβ amino acid residues 4–10, which can only recognize β-CTFs (Figure 2A). The overexpressed C99 was detected at a slightly higher molecular weight than the β-CTFs derived from endogenous or overexpressed APP due to the presence of the signal sequence and the additional residues (Dyrks et al., 1993; Lefranc-Jullien et al., 2005). To further characterize the cell lines, we evaluated the membrane localisation of APP and APP-CTFs, in particular overexpressed C99, using subcellular fractionation. Results showed that APP and all APP-CTFs, α or β, endogenous or overexpressed, are membrane-bound, as expected (Figure 2B).

**FIGURE 3:**
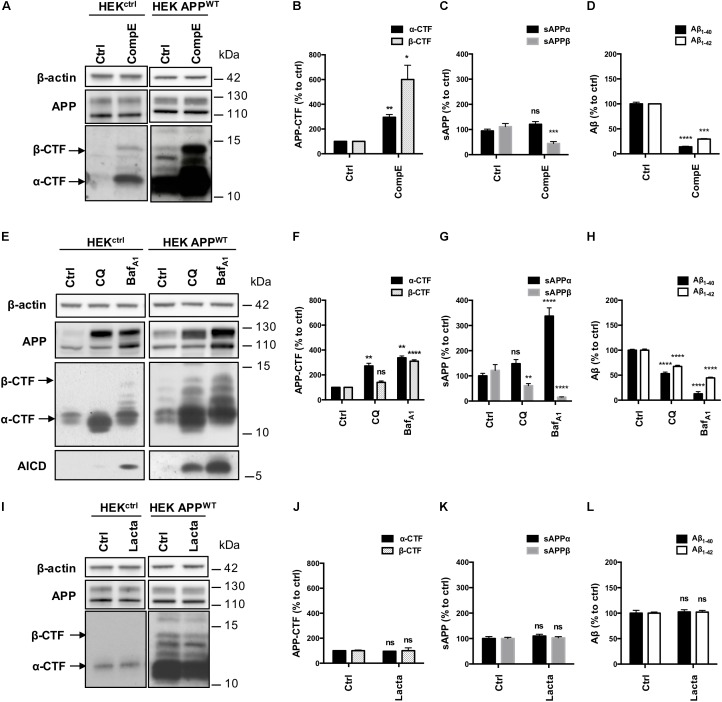
Effect of γ-secretase, lysosomal proteases or proteasome inhibition on APP processing in HEK^ctrl^ and HEK APP^WT^ cells. HEK 293 cells overexpressing or not APP^WT^ were treated with Compound E (CompE, 30 nM, 6 h), Chloroquine (CQ, 10 μM, 24 h), Bafilomycin A1 (Baf_A1_, 100 nM, 24 h) or Lactacystin (Lacta, 5 μM, 6 h). **(A,E,I)** Western-blot analysis of β-actin, total APP and APP-CTFs. β-actin staining was used as loading control. α-CTFs and β-CTFs are indicated by arrows. **(B,F,J)** Western-blot quantification of α-CTFs (black bars) and β-CTFs (dotted bars) from HEK APP^WT^ cells, expressed as percentage of the control condition. **(C,G,K)** Quantification of secreted sAPPα (black bars) and sAPPβ (gray bars) by electro-chemiluminescence immunoassay from HEK APP^WT^ cells, expressed as percentage of the control condition. **(D,H,L)** Quantification of secreted Aβ_1-40_ (black bars) and Aβ_1-42_ (white bars) measured by ELISA from HEK APP^WT^ cells, expressed as percentage of the control condition. Data are expressed as the mean ± SEM (*n* = 3 independent experiments), ^∗^*p* < 0.05, ^∗∗^*p* < 0.01, ^∗∗∗^*p* < 0.001, and ^∗∗∗∗^*p* < 0.0001. See Supplementary Figure S4 for corresponding uncropped images of western-blots.

The contribution of pathways responsible for APP-CTFs degradation was then investigated in these models using a pharmacological approach. First, HEK^ctrl^ and HEK APP^WT^ were treated for 6 h with the γ-secretase inhibitor Compound E (CompE) (Yang et al., 2008). APP and APP-CTFs levels were analyzed by Western blotting (Figure 3A). Inhibition of the γ-secretase in both HEK^ctrl^ and HEK APP^WT^ cells did not modify total APP expression (Figure 3A). In both HEK^ctrl^ and HEK APP^WT^ cells, APP-CTFs were increased after γ-secretase inhibition with α-CTF being the major species observed, indicating that overexpressed APP is processed in a similar manner as its endogenous conterpart. As the major CTFs species observed are α-CTFs in these two models, these results demonstrate that there is no bias in the amyloidogenic/non-amyloidogenic balance induced by APP overexpression. γ-secretase inhibition by CompE increased β-CTFs expression levels by 6 fold in HEK APP^WT^ (Figure 3B) while the levels of sAPPβ, Aβ_1-40_ and Aβ_1-42_ secretion, measured in the conditioned medium of the cell cultures, were reduced by 55, 85 and 70%, respectively (Figures 3C,D). These results show that γ-secretase inhibition reduced β-CTFs cleavage into Aβ peptides but surprisingly also reduced sAPPβ secretion, suggesting that γ-secretase inhibition led to a reduction of the amyloidogenic processing of APP.

Next, lysosomal proteases were inhibited using the alkalizing agent Chloroquine (CQ), a weak base, or Bafilomycin A1 (Baf_A1_), a vacuolar proton pump inhibitor. Efficiency of CQ and Baf_A1_ treatments were validated using the markers of autophagy: p62 and LC3 lipidation (Supplementary Figures S1A–C). CQ and Baf_A1_ treatments both induced a marked increase in total APP (mainly the mature form, upper band), AICD and APP-CTFs levels, especially α-CTFs in both HEK^ctrl^ and HEK APP^WT^ (Figure 3E). In HEK APP^WT^ treated cells, β-CTFs expression was increased by 40% with CQ and by 300% with Baf_A1_ (Figure 3F). Secretion of sAPPβ was reduced by 40% with CQ and by 85% with Baf_A1_ while sAPPα was significantly increased by 49 and 230%, respectively (Figure 3G). CQ and Baf_A1_ treatments also significantly repressed Aβ_1-40_ and Aβ_1-42_ secretion (Figure 3H). Treatments with CQ or Baf_A1_ inhibited β-CTFs degradation and reduced Aβ peptides and sAPPβ secretion while they increased sAPPα secretion, suggesting that the amyloidogenic pathway was reduced in favor of the α-secretase cleavage. These results are in agreement with previous reports showing that the endosomal/lysosomal pathway is of particular importance for the whole APP processing, and in particular for efficient amyloidogenic processing (Tam et al., 2016; González et al., 2017; Xu et al., 2017).

Finally, proteasome involvement was assessed using the specific inhibitor lactacystin (Lacta), which potency was validated by detection of polyubiquitinylated proteins in western-blot (Supplementary Figures S1D,E). Total APP and APP-CTFs levels were not significantly modified after 6 h of lactacystin treatment in HEK^ctrl^ or in HEK APP^WT^ treated cells (Figures 3I,J). Moreover, proteasome inhibition did not affect the secretion of sAPPα/sAPPβ or Aβ_1-40_ and Aβ_1-42_ peptides in the conditioned medium suggesting that APP processing is not modulated by the proteasome as far as our cell system is concerned (Figures 3K,L).

Together, these results demonstrate that γ-secretase and lysosomal pathway are directly involved in the processing of sAPP and APP-CTFs that derive from full-length APP, while proteasome is not involved in the degradation of these fragments in these models. Importantly, similar results were obtained both in naive HEK, expressing endogenous levels of APP, and in HEK over-expressing APP^WT^, thus showing that overexpressed APP^WT^ follows the same processing as the endogenous APP, making it a valid model to study APP processing.

### Overexpressed C99 Is Processed by γ-Secretase and the Proteasome

**FIGURE 4:**
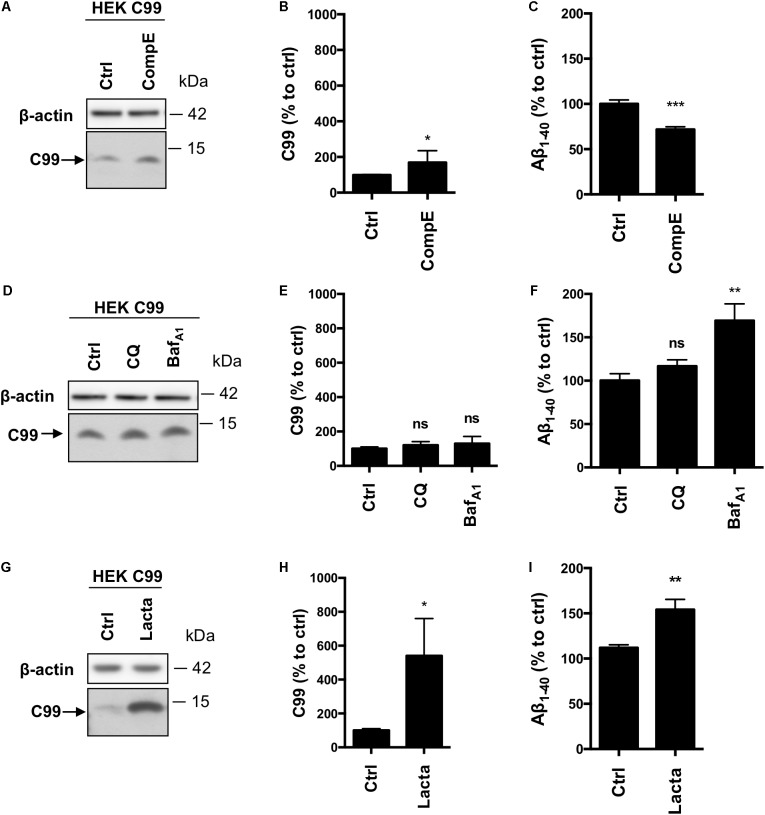
Effect of γ-secretase, lysosomal proteases or proteasome inhibition on overexpressed C99 processing in HEK^C99^ cells. HEK 293 cells overexpressing C99 (HEK^C99^) were treated with Compound E (CompE, 30 nM, 6 h), Chloroquine (CQ, 10 μM, 24 h), Bafilomycin A1 (Baf_A1_, 100 nM, 24 h) or Lactacystin (Lacta, 5 μM, 6 h). **(A,D,G)** Western-blot analysis of β-actin and C99. **(B,E,H)** Western-blot quantification of C99 from HEK^C99^, expressed as a percentage of the control condition. **(C,F,I)** ELISA quantification of secreted Aβ_1-40,_ expressed as a percentage of the control condition. Data are expressed as the mean ± SEM, (*n* = 5 independent experiments), ^∗^*p* < 0.05, ^∗∗^*p* < 0.01, and ^∗∗∗^*p* < 0.001. See Supplementary Figure S5 for corresponding uncropped images of western-blots.

Given the inconsistency between our results and the literature with regards to the involvement of the proteasome toward APP processing, in particular β-CTFs, we used HEK^C99^ cells. Indeed, most of the studies, which aim to analyze the role of the proteasome in APP processing, were performed with C99 overexpressing cells. In HEK^C99^, γ-secretase inhibition using CompE doubled the amount of C99 levels (Figures 4A,B) and reduced by 30% the secretion of Aβ_1-40_ peptides in the conditioned medium (Figure 4C). Aβ_1-42_ peptides could not be detected in these conditions, probably due to their low levels of production as described in earlier reports (Lefranc-Jullien et al., 2005). Surprisingly, in sharp contrast with results obtained in HEK APP^WT^ cells, treatments with CQ or Baf_A1_ did not significantly modify the amount of C99 (Figures 4D,E), suggesting that the lysosomal pathway is not the main degradation route for overexpressed C99. However, while CQ had no effect on Aβ_1-40_ peptides secretion, Baf_A1_ induced a 50% increase of Aβ_1-40_ peptides secretion (Figure 4F). Finally, proteasome inhibition with lactacystin induced a significant increase of C99 compared to the control condition (Figures 4G,H). Moreover, lactacystin also induced a 50% increase in Aβ_1-40_ peptides secretion (Figure 4I). To ascertain that this effect was not related to off-target effects of lactacystin, we used two other proteasomal inhibitors to validate these data. Similar results were obtained using Epoxomicin and MG132, further supporting the observation that C99 is processed by the proteasome in this model (Supplementary Figure S2A). Finally, APP overexpression was also reported to disturb proteasome activity (Matsumoto et al., 2006). To ascertain that the inhibitory effect of lactacystin on proteasome activity was conserved in HEK APP^WT^ cells, we compared the accumulation of polyubiquitinylated proteins following lactacystin treatment in the cell lines. Our data established that lactacystin treatment led to a similar increase of polyubiquitinylated proteins in all the cell lines (Supplementary Figures S2B,C), demonstrating that the differential effect of proteasome on the degradation of C99 and β-CTF cannot be attributed to an impairement of the proteasome activity in HEK APP^WT^ cells.

Taken together and in comparison to control HEK cells expressing endogenous β-CTFs (Figure 2), these results show that overexpressed C99 is mainly processed by the proteasome and to a lesser extent by γ-secretase. However, in contrast with APP-derived β-CTFs, the lysosomal pathway does not seem to be involved in the degradation of overexpressed C99. Overall, these data strongly suggest that overexpressed C99 does not follow the same degradation route as the endogenously produced APP-CTF fragments.

### Proteasomal Involvement Toward APP Processing in Primary Neuronal Cells Infected With APP^WT^ or C99

**FIGURE 5:**
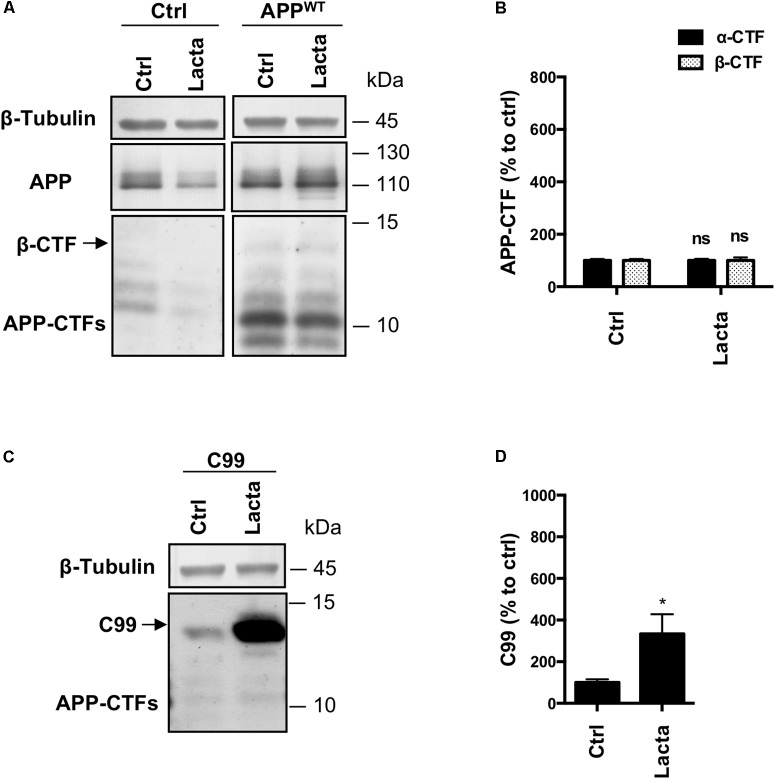
Effect of proteasome inhibition on APP-derived CTFs and C99 in primary neuronal culture cells. Rat primary neuronal cultures were treated with lactacystin (Lacta, 5 μM, 6 h) 4 days after viral infection with either APP^WT^ or C99. **(A,C)** Western-blot analysis of β-tubulin, total APP and APP-CTFs from APP^WT^ **(A)** or C99 **(C)** infected neurons. **(B,D)** Western-blot quantification of α-CTFs (black bars), β-CTFs (dotted bars) and C99 from APP^WT^ **(B)** or C99 **(D)** infected neurons, expressed as a percentage of the control condition. Data are expressed as the mean ± SEM (*n* = 3 independent experiments), ^∗^*p* < 0.05. See Supplementary Figure S6 for corresponding uncropped images of western-blots.

Finally, to exclude any cell line dependent effect, we used primary rat neuronal cells that were infected or not with APP^WT^ or C99 to validate the results regarding the proteasome involvement in β-CTFs processing in a more physiological model. Lactacystin treatment did not significantly modify APP-CTFs expression in control or APP^WT^ infected neurons (Figures 5A,B), as it was observed in HEK cells. However, overexpressed C99 was significantly accumulated in C99 infected neurons under lactacystin treatment (Figures 5C,D). Altogether, these results showed that the proteasome is not involved in the degradation of APP-derived CTFs in cultured rat neurons, whereas proteasomal inhibition strongly repressed overexpressed C99 degradation. These data are in agreement with results obtained in HEK APP^WT^ and HEK^C99^ cells, respectively.

## Discussion

APP metabolism is a key feature in the onset and progression of AD as it is at the origin of β-CTFs accumulation and Aβ peptides production. Drugs that improve the clearance of APP metabolites would possibly protect from the detrimental effects of APP-CTFs and Aβ peptides accumulation. Therefore, a better understanding of alternative degradation pathways and molecular factors modulating β-CTFs accumulation is of interest and could uncover potential interesting pharmacological targets.

**FIGURE 6:**
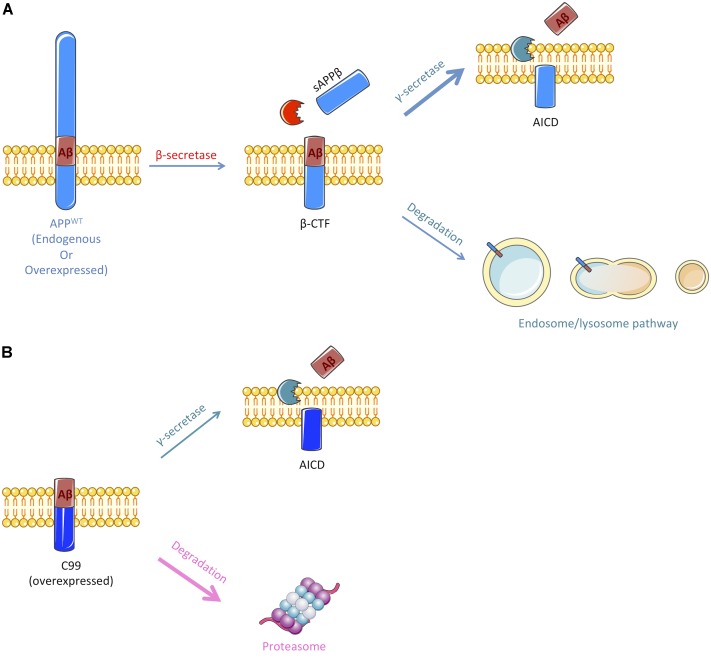
Schematic representation of the pathways implicated in the cleavage or degradation of endogenous APP-derived β-CTF or overexpressed C99. **(A)** β-CTF produced from β-secretase cleavage of full-length APP is mainly cleaved by the γ-secretase to produce Aβ peptides (nearly 60%). Remaining β-CTFs (40%) are degraded by the endosomal/lysosomal pathway. **(B)** Overexpressed C99 can also be cleaved by the γ-secretase to produce Aβ peptides, but only 30% are processed through this pathway. The remaining 70% are degraded by an alternate proteasome-dependant pathway. Figure was produced in part using Servier Medical Art.

Herein, we showed that APP-CTFs produced from endogenous or overexpressed full-length APP are mainly processed by γ-secretase and the endosomal/lysosomal pathway (observations are summarized in Figure 6A). Interestingly, γ-secretase inhibition did not only led to a reduction of Aβ peptides secretion but also to a decreased secretion of sAPPβ. This is, to the best of our knowledge, the first report suggesting that γ-secretase inhibition also repress to some extend β-secretase cleavage of APP. The mechanism by which this cross-inhibition occur will require further investigations. It could be speculated that γ-secretase substrates may include proteins that could be important for the targeting of BACE or the acidification of intracellular compartments; or that inhibiting γ-secretase activity could reroute APP toward an alternative β-secretase activity, i.e., meprin β, thus producing sAPPβ that would not be recognized by the electro-chemiluminescence immunoassay kit used in this study.

Our results also confirmed that besides being cleaved by γ-secretase, APP-CTFs are efficiently degraded by the endosomal/lysosomal pathway, as it was widely reported (Tam et al., 2016; González et al., 2017; Xu et al., 2017). Along with reducing APP-CTFs degradation, drugs that were used to inhibit the endosomal/lysosomal pathway significantly reduced the secretion of Aβ peptides and sAPPβ species. As we previously showed that the inhibition of the endosomal/lysosomal pathway does not alter γ-secretase activity (Vingtdeux et al., 2007a), these results suggest that there could be an impairment of the extracellular release of Aβ and sAPPβ from recycling compartments or that the β-secretase cleavage of APP is inhibited by these lysomotropic agents. The cleavage of APP by BACE1 occurs in acidic endosomal compartments, with an optimal activity at pH 4.5 (Vassar et al., 1999; Sun and Roy, 2017). Thus, it is not surprising that these drugs, which act by reducing the acidification of the endosomal and lysosomal compartments, would lead to an inhibition of BACE1-mediated APP processing and a parallel increase of sAPPα secretion, which could result from a redirection of APP to the non-amyloidogenic pathway. The development of drugs/molecules that derive from the CQ structure and share its effects on APP processing is an interesting strategy that is currently developed to tackle AD (Melnyk et al., 2015). Interestingly, we recently described the identification of CQ derivatives that retained their effects toward Aβ secretion without impacting APP-CTFs accumulation through lysomotropic activity (Gay et al., 2018).

In sharp contrast to APP derived β-CTFs degradation, we found that overexpressed C99 was mainly degraded by the proteasome and to a lower extent by γ-secretase (Figure 6B). Indeed, lactacystin led to a strong accumulation of overexpressed C99 that was accompanied by an increase in Aβ_1-40_ peptides secretion. The strong accumulation of C99 following proteasome inhibition could lead to an upsurge in γ-secretase substrate availability and therefore to an increase in Aβ peptides production, without affecting γ-secretase activity. However, we cannot exclude the possibility that γ-secretase activity could be altered following proteasome inhibition. Indeed, components of the γ-secretase complex have been shown to be ubiquitinylated and thus suggested to be handled by the proteasome (Kim et al., 1997; Marambaud et al., 1998; He et al., 2006, 2007).

The different processing of overexpressed C99 as compared to APP-derived β-CTFs by the proteasome could be an artefactual consequence of the overexpression. Indeed, several reports have shown that overexpressed proteins can be processed in an artefactual way by the proteasome (Dunys et al., 2006). Dunys et al. also proposed that lactacystin could activate CMV promoters thus, leading to an artefactual increase in expression levels. However, expression of full-length APP, which was also overexpressed in our HEK APP^WT^ model, was not affected by lactacystin treatment. In addition, two other proteasome inhibitors also led to C99 increase. The most straightforward conclusion here is that, when ectopically over-expressed, C99 is recognized as a truncated membrane protein that will undergo ER-associated degradation (ERAD) mediated by the proteasome (Avci and Lemberg, 2015; Jang et al., 2017). This is not the case for APP-CTFs that are endogenously produced along the endosome/lysosome pathway. However, it will be interesting to determine whether mutations in APP that are associated with familial forms of AD could affect APP processing by the endosomal/lysosomal or proteasomal pathway.

In conclusion, our data show that APP-derived CTFs are processed principally by the γ-secretase and alternatively degraded by lysosomes. Indeed, the physiological processing of APP by α- or β-secretase relies on the endosomal/lysosomal pathway, which dysfunction leads to a complete disturbance of APP metabolites. However, a direct degradation of APP-derived CTFs by a proteasome-dependent pathway is not supported experimentally in this study. In sharp contrast, C99 chimeric construct is mainly processed by a proteasome-dependent mechanism and to a lesser extent by γ-secretase. Hence, homologies between APP-derived β-CTFs and overexpressed C99 processing are likely restricted to their processing by γ-secretase, therefore caution should be taken when using this model to study β-CTFs biology.

## Author Contributions

PK-C, J-NO, LB, NS, and VV contributed conception and design of the study. CE, PK-C, MC, RO, BT, and VV carried out the experiments. CE performed the statistical analysis. PM provided study materials. CE, NS, and VV wrote the manuscript. All authors contributed to manuscript revision, read and approved the submitted version.

## Conflict of Interest Statement

The authors declare that the research was conducted in the absence of any commercial or financial relationships that could be construed as a potential conflict of interest.

## Supplementary Material

FIGURE S1 Validation of inhibitors efficacy. HEK 293 cells were treated with Chloroquine (CQ, 10 μM, 24 h), Bafilomycin A1 (Baf_A1_, 100 nM, 24 h) or Lactacystin (Lacta, 5 μM, 6 h). **(A,D)** Western-blot analysis of β-actin, p62, LC3-B and polyubiquitinylated proteins. β-actin staining was used as loading control. **(B,C,E)** Western-blot quantification of p62, LC3-II/LC3-I ratio and poly ubiquitin from HEK APP^WT^ cells, expressed as a percentage of the control condition. Inhibition of lysosomal flux was validated by the increase of two markers associated with autophagy: p62 and LC3-I lipidation into LC3-II and inhibition of proteasomal degradation was validated by the accumulation of polyubiquitinylated proteins in cells. Data are expressed as the mean ± SEM (*n* = 3 independent experiments), ^∗∗^*p* < 0.01, ^∗∗∗^*p* < 0.001, and ^∗∗∗∗^*p* < 0.0001.

FIGURE S2 Representative effects of other proteasome inhibitors on overexpressed C99 processing in HEK^C99^ cells and proteasome activity in the different HEK 293 cell lines used in this study. **(A)** Western-blot analysis of β-actin and C99 in HEK^C99^ treated with Expoxomicin (Epoxo, 1 μM, 6 h) or MG132 (10 μM, 6 h). β-actin staining was used as loading control (*n* = 3 independent experiments). **(B)** Western-blot analysis of β-actin and polyubiquitinylated proteins expression in naive HEK 293 cells (HEK^ctrl^), and HEK 293 overexpressing full length APP^WT^ (HEK APP^WT^) or C99 (HEK^C99^) cells treated with Lactacystin (Lacta, 5 μM, 6 h) or not (Ctrl). β-actin staining was used as loading control. **(C)** Western-blot quantification of polyubiquitinylated proteins, expressed as percentage of the control condition. Data are expressed as mean ± SEM (*n* = 3 independent experiments), ^∗∗^*p* < 0.01 using the unpaired Student’s test for pairwise comparisons between control and lactacystin conditions. Using the one way ANOVA with Bonferroni’s multiple comparisons test for comparisons between cell lines, no significant diffence was observed.

FIGURE S3 Uncropped images of western-blot presented in Figure 2.

FIGURE S4 Uncropped images of western-blot presented in Figure 3.

FIGURE S5 Uncropped images of western-blot presented in Figure 4.

FIGURE S6 Uncropped images of western-blot presented in Figure 5.
