# Supplementary figures and images for: Contribution of the Endosomal-Lysosomal and Proteasomal Systems in Amyloid-β Precursor Protein Derived Fragments Processing
Source: Front Cell Neurosci. 2018 Nov 22;12:435. doi: 10.3389/fncel.2018.00435 (PMC6263093; doi:10.3389/fncel.2018.00435)

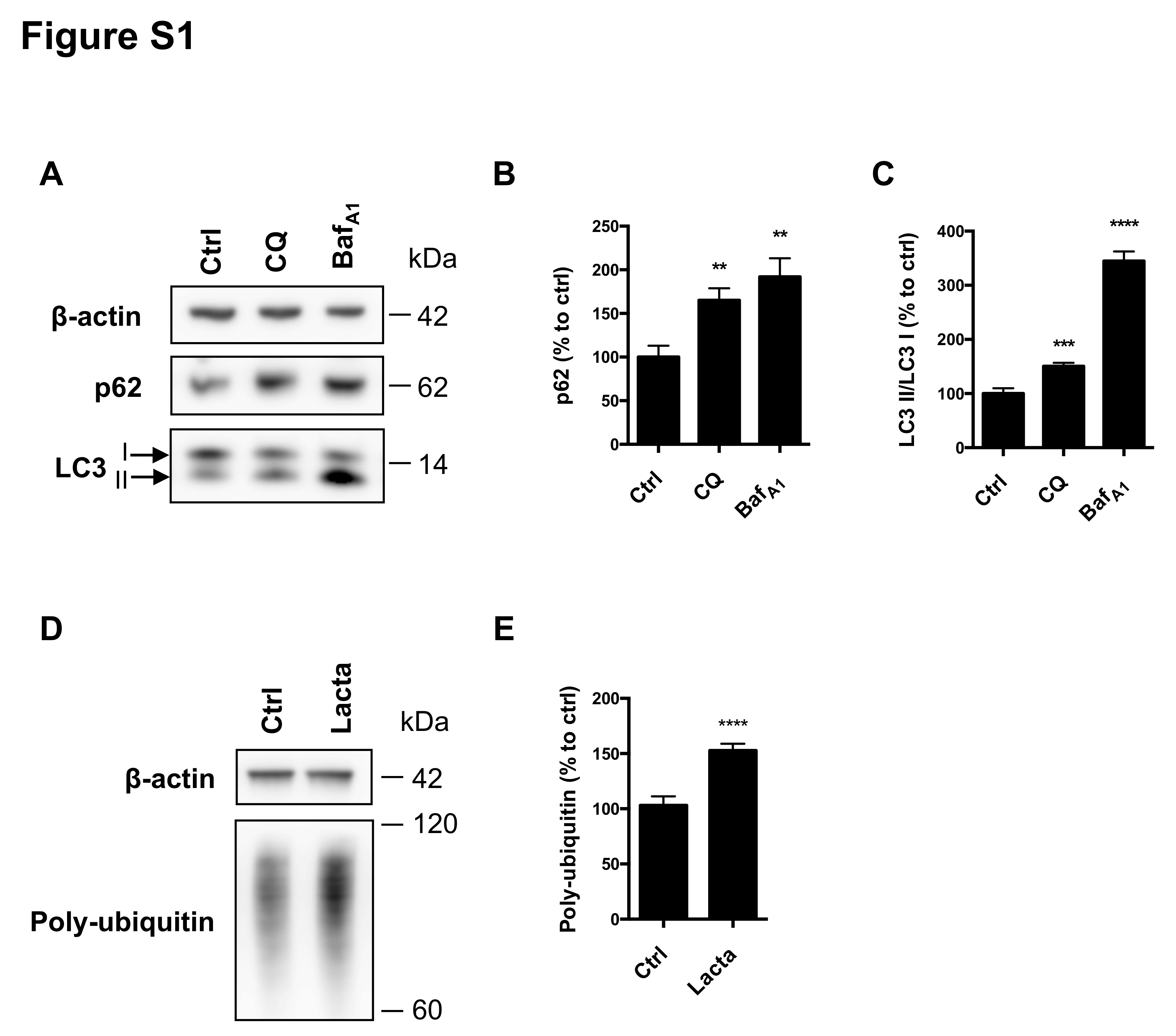

Supplement: FIGURE S1 — Validation of inhibitors efficacy. HEK 293 cells were treated with Chloroquine (CQ, 10 μM, 24 h), Bafilomycin A1 (BafA1, 100 nM, 24 h) or Lactacystin (Lacta, 5 μM, 6 h). (A,D) Western-blot analysis of β-actin, p62, LC3-B and polyubiquitinylated proteins. β-actin staining was used as loading control. (B,C,E) Western-blot quantification of p62, LC3-II/LC3-I ratio and poly ubiquitin from HEK APPWT cells, expressed as a percentage of the control condition. Inhibition of lysosomal flux was validated by the increase of two markers associated with autophagy: p62 and LC3-I lipidation into LC3-II and inhibition of proteasomal degradation was validated by the accumulation of polyubiquitinylated proteins in cells. Data are expressed as the mean ± SEM (n = 3 independent experiments), ∗∗p < 0.01, ∗∗∗p < 0.001, and ∗∗∗∗p < 0.0001. [file Image_1.TIF]

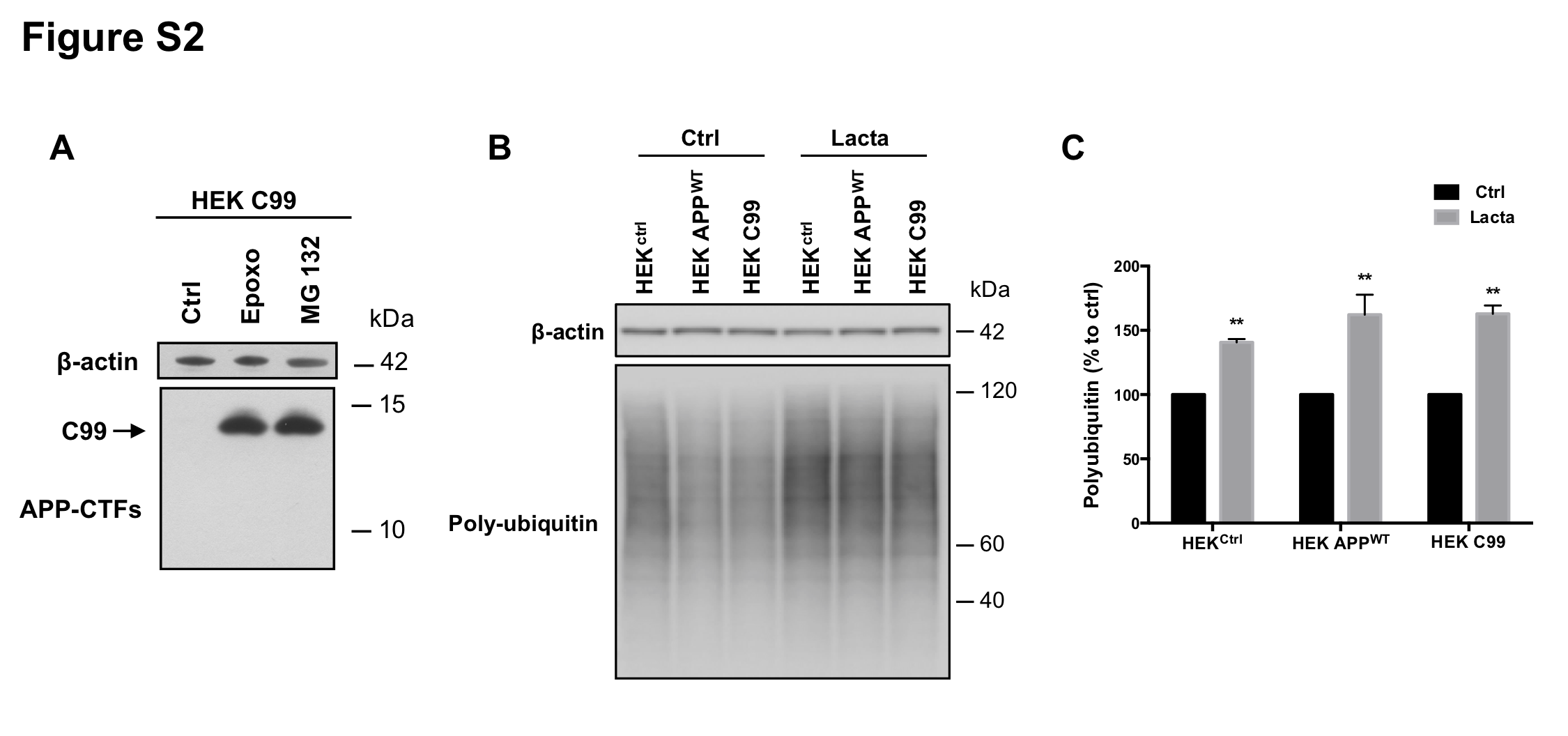

Supplement: FIGURE S2 — Representative effects of other proteasome inhibitors on overexpressed C99 processing in HEKC99 cells and proteasome activity in the different HEK 293 cell lines used in this study. (A) Western-blot analysis of β-actin and C99 in HEKC99 treated with Expoxomicin (Epoxo, 1 μM, 6 h) or MG132 (10 μM, 6 h). β-actin staining was used as loading control (n = 3 independent experiments). (B) Western-blot analysis of β-actin and polyubiquitinylated proteins expression in naive HEK 293 cells (HEKctrl), and HEK 293 overexpressing full length APPWT (HEK APPWT) or C99 (HEKC99) cells treated with Lactacystin (Lacta, 5 μM, 6 h) or not (Ctrl). β-actin staining was used as loading control. (C) Western-blot quantification of polyubiquitinylated proteins, expressed as percentage of the control condition. Data are expressed as mean ± SEM (n = 3 independent experiments), ∗∗p < 0.01 using the unpaired Student’s test for pairwise comparisons between control and lactacystin conditions. Using the one way ANOVA with Bonferroni’s multiple comparisons test for comparisons between cell lines, no significant diffence was observed. [file Image_2.TIF]

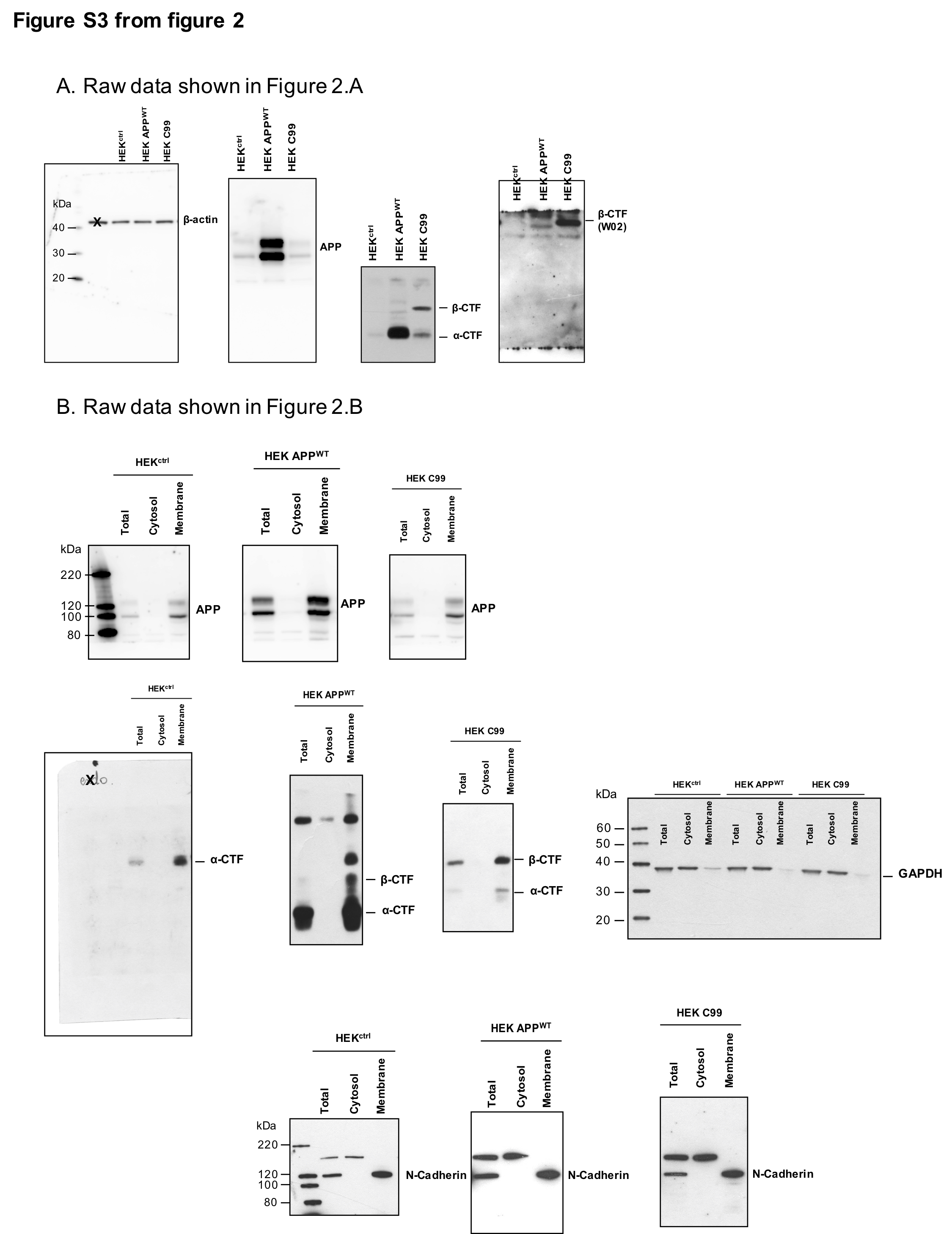

Supplement: FIGURE S3 — Uncropped images of western-blot presented in Figure 2. [file Image_3.TIF]

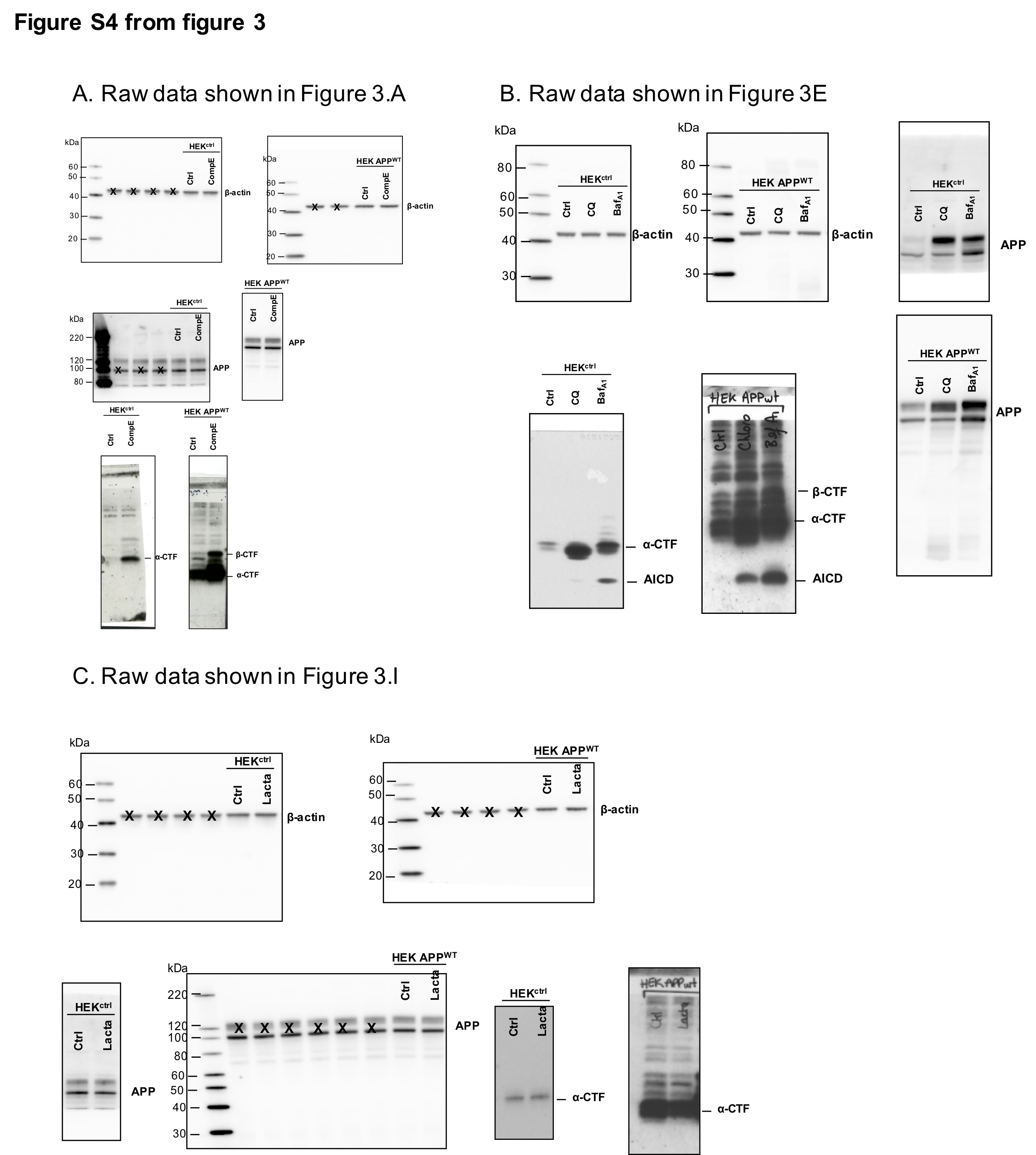

Supplement: FIGURE S4 — Uncropped images of western-blot presented in Figure 3. [file Image_4.TIF]

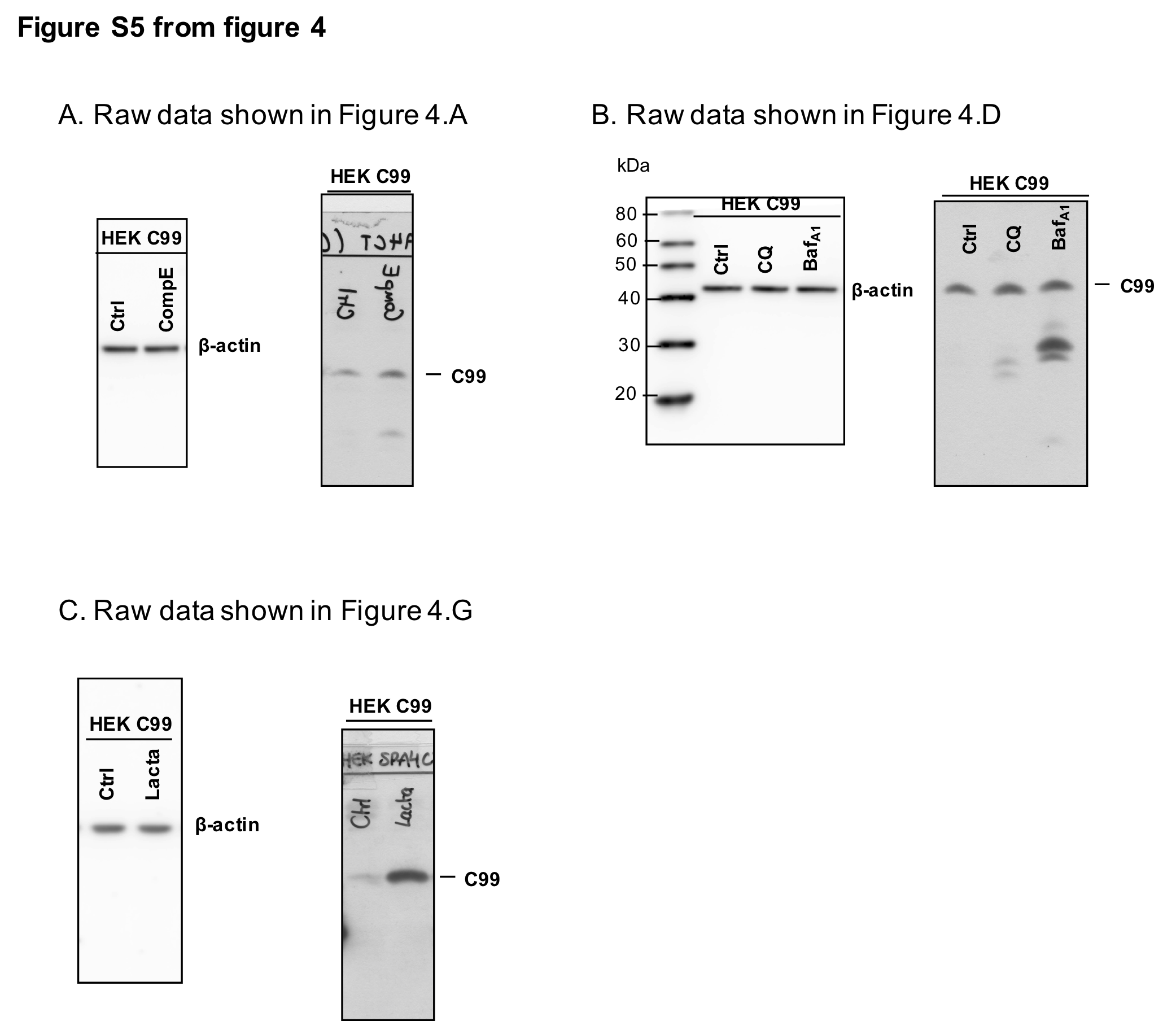

Supplement: FIGURE S5 — Uncropped images of western-blot presented in Figure 4. [file Image_5.TIF]

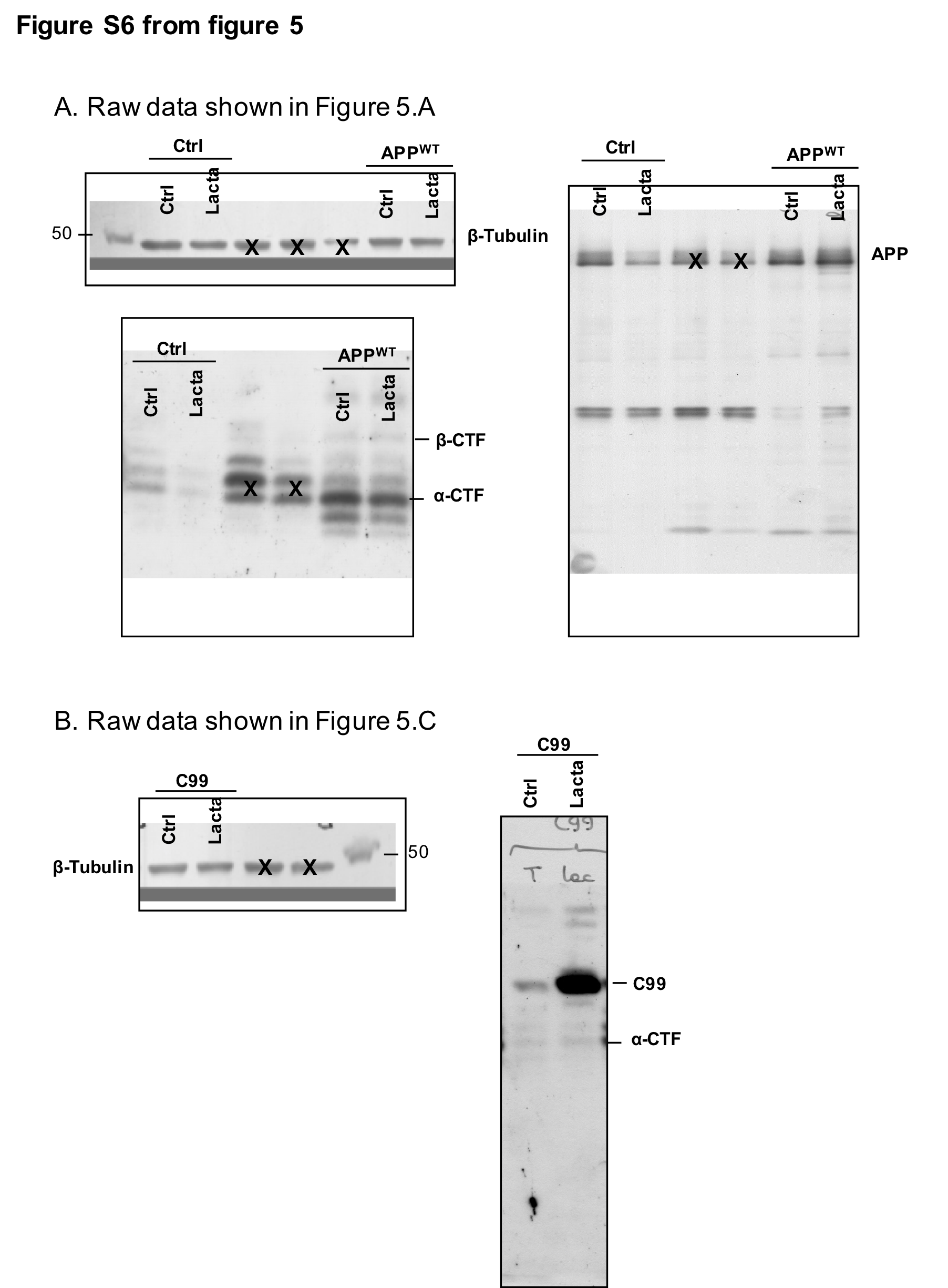

Supplement: FIGURE S6 — Uncropped images of western-blot presented in Figure 5. [file Image_6.TIF]
